# Tuberculosis of the Appendix

**Published:** 1905-06-03

**Authors:** 


### TUBERCULOSIS OF THE APPENDIX.

When an individual, known to be the subject of pulmonary tuberculosis, develops symptoms pointing to an inflammatory condition of the appendix a fear not unnaturally arises lest tuberculous involvement of that organ may underlie the symptoms. Tuberculosis of the intestines is, of course, a frequent complication of the pulmonary disease, and in adults the region of the cæcum is more prone, perhaps, than other parts of the alimentary canal to show evidence of the infection. But there has been little collected information as to the probabilities of tuberculosis in cases clinically of the ordinary appendicular type. Dr. Maurice Letulle[1] provides some useful grounds for deduction in the matter as the result of a microscopic examination of some 300 appendices removed by operation. In the whole series he found only two cases where the appendix was tuberculous, and only one of these could be fairly considered a case of the ordinary type. The details of this case are as follows:—The patient was a young man of 26 resident in a sanatorium for the treatment of pulmonary tuberculosis. During a five months' residence he had five acute attacks of appendicitis, each accompanied by much vomiting. The probability that tuberculosis underlay the condition prompted postponement of operative interference, which was ultimately undertaken under protest and at the urgent instance of the patient. The appendix, though somewhat adherent, was removed without difficulty and submitted to microscopic examination. The result was that Dr. Letulle was able clearly to recognise the existence of a double infection, for while the mucosa was extensively caseated, at other parts, where the tissues had escaped the tuberculous process, there was good evidence of an acute inflammation in the presence of copious small-celled infiltration. Among the author's conclusions the following is the most important: — That a tuberculous lesion of the appendix, which is usually ulcerative, appears not to favour the development of acute secondary infections capable of producing an attack of typical non-tuberculous appendicitis.

[1] Revue de la Tuberculose, Feb. 1905.

